# Vascular Normalization Caused by Short-Term Lenvatinib Could Enhance Transarterial Chemoembolization in Hepatocellular Carcinoma

**DOI:** 10.3390/curroncol30050360

**Published:** 2023-05-05

**Authors:** Tetsuya Tachiiri, Hideyuki Nishiofuku, Shinsaku Maeda, Takeshi Sato, Shohei Toyoda, Takeshi Matsumoto, Yuto Chanoki, Kiyoyuki Minamiguchi, Ryosuke Taiji, Hideki Kunichika, Satoshi Yamauchi, Takahiro Ito, Nagaaki Marugami, Toshihiro Tanaka

**Affiliations:** Department of Diagnostic and Interventional Radiology, Nara Medical University, Kashihara 634-8522, Japan; hmn@naramed-u.ac.jp (H.N.); shinsaku@naramed-u.ac.jp (S.M.); satotake@naramed-u.ac.jp (T.S.); s.toyoda@naramed-u.ac.jp (S.T.); t.matsumoto@naramed-u.ac.jp (T.M.); y.chanoki@naramed-u.ac.jp (Y.C.); kiyo829@naramed-u.ac.jp (K.M.); rtaiji1119@gmail.com (R.T.); k102972@naramed-u.ac.jp (H.K.); syamauchi@naramed-u.ac.jp (S.Y.); titoh@naramed-u.ac.jp (T.I.); marugami@naramed-u.ac.jp (N.M.); totanaka@naramed-u.ac.jp (T.T.)

**Keywords:** hepatocellular carcinoma, vascular normalization, lenvatinib, multi-kinase inhibitor, transarterial chemoembolization, perfusion imaging

## Abstract

We describe the clinical effects of short-term lenvatinib administration prior to conventional transarterial chemoembolization (cTACE) on tumor vasculature. Two patients with unresectable hepatocellular carcinoma underwent high-resolution digital subtraction angiography (DSA) and perfusion four-dimensional computed tomography during hepatic arteriography (4D-CTHA) before and after administration of lenvatinib treatment. The doses and periods of lenvatinib administration were, respectively, 12 mg/day for 7 days and 8 mg/day for 4 days. In both cases, high-resolution DSA revealed a decrease in dilatation and tortuosity of the tumor vessels. Furthermore, the tumor staining became more refined, and newly formed tiny tumor vessels were observed. Perfusion 4D-CTHA revealed a decrease in arterial blood flow to the tumor by 28.6% (from 487.9 to 139.5 mL/min/100 mg) and 42.5% (from 288.2 to 122.6 mL/min/100 mg) in the two cases, respectively. The cTACE procedure resulted in good lipiodol accumulation and complete response. Patients have remained recurrence-free for 12 and 11 months after the cTACE procedure, respectively. The administration of short-term lenvatinib in these two cases resulted in the normalization of tumor vessels, which likely led to improved lipiodol accumulation and a favorable antitumor effect.

## 1. Introduction

Transarterial chemoembolization (TACE) is a standard treatment for intermediate-stage hepatocellular carcinoma (HCC) [1]. However, the effect of TACE alone is limited and new effective therapeutic strategies are urgently needed [2,3]. In the past several years, clinical trials assessing the combination of TACE plus antiangiogenic agents have been designed [4,5].

Lenvatinib [6] is an orally acting multi-kinase inhibitor approved by the Food and Drug Administration that targets vascular endothelial growth factor (VEGF) receptors, fibroblast growth factor receptors (FGFR), platelet-derived growth factor receptor-alpha (PDGFRα), and RET and KIT proto-oncogenes. After the SHARP trial (2008) [7], sorafenib was the only systemic therapy with proven efficacy in the treatment of patients with advanced hepatocellular carcinoma. In the REFLECT trial (2018) [8], lenvatinib demonstrated a similar overall survival but improved progression-free survival.

Recently, a Phase II clinical trial of TACE plus lenvatinib (TACTICS-L) demonstrated a high complete response (CR) rate of 67.7%, as well as a high objective response rate of 75.8%, and long progression-free and overall survivals [9]. This considerable anti-tumor effect could be explained by the synergistic anticancer activity of TACE and lenvatinib.

Previously published literature has stated that antiangiogenic therapies improve the structure and function of tumor vessels [10,11]. Theoretically, this vascular normalization induces an increase in vascular perfusion, which might improve drug delivery in cancer cells [12,13]. In an animal study, lenvatinib increased pericyte coverage of tumor vessels, as well as the number of perfusing vessels, and decreased interstitial fluid pressure [14]. These results could explain the improved distribution of intra-arterial drugs administered after lenvatinib.

On the other hand, high-dose and/or long-term antiangiogenic therapy is known to cause large-scale vascular pruning [15,16,17]. The anti-angiogenic effect of lenvatinib might induce tumor starvation and suppress the effect of TACE if administered improperly. Therefore, it is important to understand the optimal dosing period of lenvatinib administered before TACE.

Although the combination of lenvatinib and TACE is a promising therapy, the optimal administration protocol of lenvatinib before TACE has not been determined. To the best of our knowledge, no previous report has demonstrated vascular normalization with anti-angiogenic drugs in clinical angiography. Additionally, there is no research study discussing the optimal dosing period of lenvatinib administered in combination with TACE. Herein, we present two cases in which high-resolution digital subtraction angiography (DSA) and perfusion four-dimensional computed tomography during hepatic arteriography (4D-CTHA) images were obtained before and after short-term lenvatinib administration, following which TACE was successfully performed. In this report, we discuss the clinical effects of short-term lenvatinib administration prior to conventional transarterial chemoembolization (cTACE) on tumor vasculature. From these findings, we will propose that short-term lenvatinib prior to cTACE could become a standardized procedure in unresectable HCC.

At our institution, institutional review board (IRB) approval is not required for case reports. Written informed consent was obtained from each patient before treatment.

## 2. Case Presentations

### 2.1. Case 1

The patient was a 73-year-old man with alcoholic hepatitis. He received conventional TACE (cTACE) and hepatic arterial infusion chemotherapy (HAIC) combined with radiotherapy (RT) for HCC with tumor thrombus in the right portal vein. Subsequently, systemic therapy of atezolizumab plus bevacizumab was started. Eight months later, HCC recurrence in segment (S) 5 (18 mm in diameter) and S6 (8 mm in diameter) was identified on contrast-enhanced (CE) CT. The liver function was Child–Pugh 5A. Superselective TACE was planned for the two lesions.

Since hepatic angiography obtained before TACE revealed multiple tiny tumor stains in the right hepatic lobe, we suspected other additional recurrences. Therefore, we suspended performing TACE and instead performed DSA using a high-resolution angiography system (Alphenix Sky+, Canon Medical Systems Corporation, Otawara, Japan). Additionally, CTHA was performed using a 320-slice area detector CT (Aquilion One, Canon Medical Systems Co.) for planning further treatment. The perfusion 4D-CTHA scan protocol was as follows: a bolus of 40 mL of diluted non-ionic iodinated contrast agent (Iopamidol 370 mg I/mL mixed with saline at a 3:1 ratio) was injected via the common hepatic artery at a rate of 2.0 mL/s. CT scanning was initiated at the same time as the start of the injection with the following acquisition parameters: 100 kV tube voltage; 120 mAs; 5 mm slice thickness; 320 × 0.5 mm collimation; 0.5 s rotation time; 2–4 s cycle time. We used perfusion CT software (Canon Medical Systems Co.) to acquire hepatic perfusion data, which allow the calculation of arterial blood flow (ABF).

**Figure 1:**
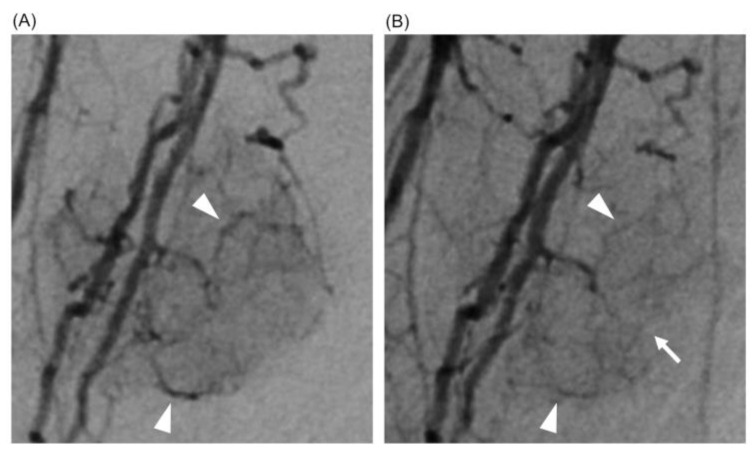
Case 1: normalization of tumor vessels in a 73-year-old man with hepatocellular carcinoma. (**A**) High-resolution DSA just before the initiation of lenvatinib. Irregular and dilated abnormal tumor vessels were seen (arrowheads). (**B**) High-resolution DSA after lenvatinib administration for 7 days. The tumor vessels became smoother and thinner (arrowheads). In addition, tumor staining became finer, and tiny tumor vessels newly appeared (white arrow).

**Figure 2:**
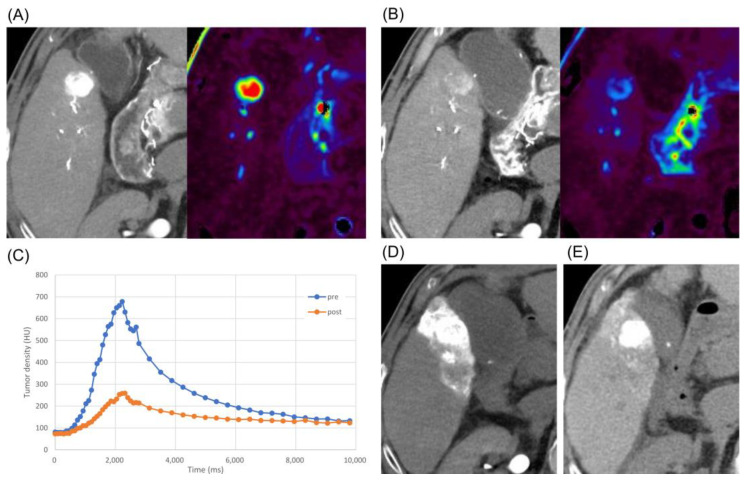
Case 1: quantification of tumor vascularity. (**A**) 4D-CTHA and perfusion map just before the initiation of lenvatinib. Arterial blood flow (ABF) to the tumor was 487.9 mL/min/100 mL. (**B**) 4D-CTHA and perfusion map after lenvatinib administration for 7 days. ABF decreased to 139.5 mL/min/100 mL, which was 28.6% of the value before lenvatinib administration. (**C**) Time intensity curves pre- and post-lenvatinib: a decrease in intratumoral density was observed post-treatment compared to pre-treatment. (**D**) Plain CT immediately after cTACE: lipiodol deposited well in the tumor. (**E**) Plain CT after 6 months: persistent complete lipiodol retention was seen in the tumor.

Subsequently, we determined a change in the therapeutic strategy, combining lenvatinib–TACE therapy. Lenvatinib at a dose of 12 mg was administered for 7 days, and high-resolution DSA and perfusion 4D-CTHA were repeated on the 8th day after the initiation of lenvatinib. A comparison of the images obtained before and after lenvatinib administration for 7 days showed that the irregular and dilated tumor vessels had become smoother and thinner. Further, tumor staining became more refined and tiny tumor vessels newly appeared, confirming normalization of the tumor vessels (Figure 1). On perfusion 4D-CTHA, the arterial blood flow (ABF) into the tumor had decreased to 28.6% of the pre-lenvatinib value (pre: 487.9 to post: 139.5 mL/min/100 mL) (Figure 2).

Next, cTACE, using an emulsion of 1.7 mL lipiodol (Guerbet, Villepinte, France) and 13 mg/0.8 mL epirubicin solution, followed by gelatin sponge particles (Gelpart, Nippon Kayaku, Tokyo, Japan), was performed. As a result, lipiodol accumulated well in tumors. The patient experienced grade 1 nausea and fever according to the National Cancer Institute Common Terminology Criteria for Adverse Events (CTCAE) ver. 5.0. Additionally, the patient exhibited grade 2 AST elevation on the day following TACE. Currently, 12 months after cTACE, there has been no tumor recurrence.

### 2.2. Case 2

The patient was a 72-year-old male with a history of liver cirrhosis due to hepatitis C virus. At the age of 68 years, the patient had undergone radiofrequency ablation for solitary HCC. Two years later, two sessions of cTACE were performed for recurrence at a previous hospital. Thereafter, he had defaulted from follow-up. He visited our hospital one year later, intending to continue treatment for HCC. CE-CT revealed HCC recurrence in S4 (59 mm in diameter) and S8 (40 mm in diameter). His liver function was Child–Pugh 5A.

Prior to performing TACE, hepatic angiography revealed a significant arterioportal shunt in the S4 tumor due to portal vein invasion. As a result, we determined that TACE was not a viable treatment option for the S4 tumor and that an alternative therapeutic strategy was necessary. We obtained high-resolution DSA and perfusion 4D-CTHA with the same scan protocol as in Case 1.

**Figure 3:**
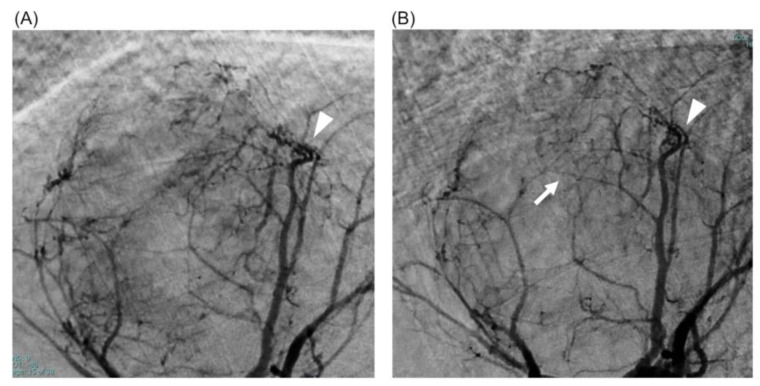
Case 2: normalization of tumor vessels in a 72-year-old man with hepatocellular carcinoma. (**A**) High-resolution DSA just before the initiation of lenvatinib. Dilated abnormal tumor vessels were seen (arrowhead). (**B**) High-resolution DSA after lenvatinib administration for 4 days. The tumor vessels became less dilated and less tortuous (arrowhead), and tiny tumor vessels newly appeared (white arrow).

**Figure 4:**
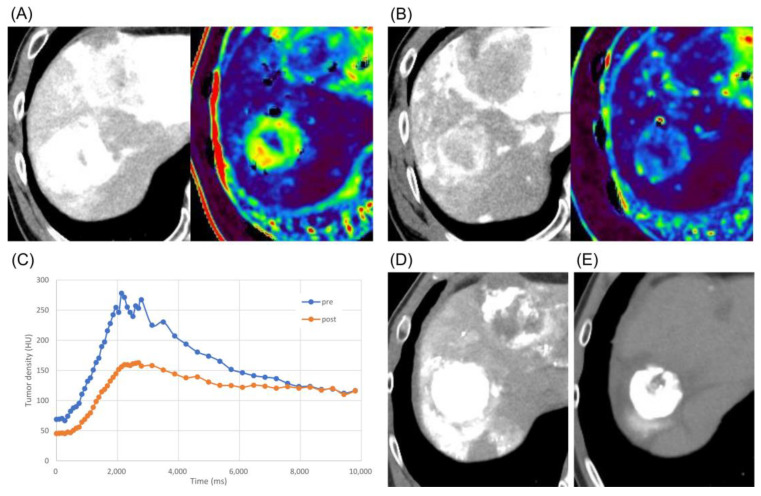
Case 2: quantification of tumor vascularity. (**A**) 4D-CTHA and perfusion map just before the initiation of lenvatinib. Arterial blood flow (ABF) to the tumor was 288.2 mL/min/100 mL. (**B**) 4D-CTHA and perfusion map after lenvatinib administration for 4 days. The ABF decreased to 122.6 mL/min/100 mL, which was 42.5% of the value before lenvatinib administration. (**C**) Time intensity curves pre- and post-treatment: a decrease in intratumoral density was observed post-treatment compared to pre-treatment. (**D**) Plain CT immediately after cTACE: lipiodol accumulated well in the tumor. (**E**) Plain CT after 5 months: the tumor had decreased in size with persistent complete lipiodol retention in the lesion.

The alternative therapeutic strategy was as follows: after lenvatinib was initially administered, TACE and HAIC were performed for the tumors in S8 and S4, respectively. Then, lenvatinib at a dose of 8 mg was administered for 4 days. High-resolution DSA and perfusion 4D-CTHA were repeated on the 5th day after the initiation of lenvatinib, with the same protocol as before. Comparison of the S8 tumor images before and after 4 days of lenvatinib administration showed that the tumor vessels had become less dilated and less tortuous, and that tiny tumor vessels had newly appeared (Figure 3). On perfusion 4D-CTHA, the ABF of the tumor had decreased to 42.5% of the pre-treatment value (pre: 288.2 to post: 122.6 mL/min/100 mL) (Figure 4).

cTACE using an emulsion of 5.3 mL lipiodol and 33 mg/2.7 mL epirubicin solution, followed by gelatin sponge particles, was performed for the S8 tumor. The lipiodol accumulated well in the tumor. The patient experienced grade 1 nausea/anorexia, fever, and tenderness in the right upper abdomen. Additionally, the patient exhibited Grade 4 AST and ALT elevations that were observed the day after the TACE procedure. However, these elevations improved over the next seven days. Currently, 11 months after cTACE, there has been no tumor recurrence. For the tumor in S4, HAIC using a cisplatin solution (IA call, Nippon Kayaku) was performed during lenvatinib administration, which resulted in good control of the tumor.

## 3. Discussion

In both our cases, we confirmed a decrease in dilatation and tortuosity of the tumor vessels on angiography after lenvatinib administration. To the best of our knowledge, this is the first report clinically showing vascular normalization induced by antiangiogenic drugs. We used a newly developed angiography system with a 76-micron pixel high-definition detector with more than twice the spatial resolution of conventional detectors. Our two cases also depicted a reduction in tumor stains and the appearance of tiny new tumor vessels. These findings can be explained by a decrease in the permeability of tumor vessels [10,11,12,13]. In general, abnormal tumor vessels are associated with leakage of blood. Normalized vasculature, on the other hand, has more normal basement membranes with higher pericyte coverage [12,13]. In our cases, a decrease in the leakage of contrast material probably led to the appearance of tiny tumor vessels in post-treatment angiography.

In addition, perfusion 4D-CTHA demonstrated a decrease in arterial tumor perfusion after lenvatinib therapy. The theory of multi-step changes in intratumoral arterial supply during hepatocarcinogenesis is well known [18,19]. In the early stages of hepatocarcinogenesis, normal arterial supply decreases and the subsequent expression of angiogenetic factors, such as VEGF, induces growth of abnormal vessels [20,21]. Lenvatinib decreases abnormal vessels and increases normal vessels by its antiangiogenic effects [13], with a consequent decrease in tumor vascularity. Based on this theory, it could be considered that a decrease in tumor vascularity indicates the normalization of abnormal vessels.

Several previous reports evaluated changes in tumor vascularity by CE ultrasound (CE-US) as early imaging biomarkers of lenvatinib efficacy [22,23]. The drawback of CE-US is that it is an operator-dependent examination compared with CT or MRI. We used a 320-row area detector CT, which can obtain 4D images of 16 cm in length with high rotational speed in our patients.

To date, optimal administration and rest durations of lenvatinib before TACE have not been determined. Kuroda et al. reported lenvatinib administration for 8 months, followed by 2 days of rest [24]. Ueshima et al. reported 14 to 21 days of administration followed by 2 days of rest (TACTICS-L trial) [8]. On the other hand, it is known that a decrease in tumor blood flow is observed within the first 7 days of lenvatinib administration, as evaluated by CE-US [22,23]. In our two cases, we performed TACE after lenvatinib administration for 7 and 4 days, respectively, with no rest period. Our study found that lenvatinib administration resulted in normalized vasculature and reduced arterial perfusion of the tumor. These results are consistent with previous studies using contrast-enhanced ultrasound, which also showed a decrease in tumor perfusion values of 41% to 56% after one week of lenvatinib administration.

It is possible that the prolonged administration of lenvatinib may lead to tumor hypoxia and starvation of tumor vessels, making the TACE procedure difficult [9]. Additionally, long-term lenvatinib use could cause adverse effects such as fatigue or loss of appetite [25]. Therefore, short-term administration may be appropriate before TACE therapy. In this study, patients receiving lenvatinib plus TACE combination therapy experienced certain side effects, including fever, nausea, anorexia, tenderness, as well as elevated levels of AST and ALT [26]. However, no increased likelihood of adverse events was observed when compared to patients who received cTACE alone.

It is well known that serum VEGF increases 1–2 days after TACE [27]. A previously published study demonstrated that overall survival of patients in the high VEGF group was shorter [28]. Therefore, theoretically, the rest duration of anti-VEGF therapy before TACE should be shorter. In our two cases, lenvatinib was administered without the rest period.

Theoretically, normalized vessels with pericyte coverage decrease leakage of blood and interstitial high fluid pressure, which might lead to improved delivery of systemically anticancer drugs or macromolecules and to improved lipiodol emulsion delivery in the tumor [10,11,12,13]. In both our cases, there were dense lipiodol accumulations in the tumors, and there has currently been no recurrence after the treatment. This combination therapy can be applied not only to TACE but also to other locoregional therapies, i.e., radiofrequency ablation or cryoablation [29].

The limitations of this study are its short follow-up period and the number of patients. In order to fully comprehend the long-term effects and benefits of this procedure, a more extensive follow-up of a larger number of patients is necessary. Additionally, the staining effect of lipiodol can result in an underestimation of the amount of tumor tissue remaining after cTACE when using CT scans. Thus, the use of MRI would be more desirable.

## 4. Conclusions

This study demonstrated the radiographic evidence of normalized tumor vessels caused by lenvatinib administration for 4 days and 7 days, respectively. Consequently, following cTACE, dense accumulations of lipiodol within the tumors were achieved without recurrence for 12 and 11 months, respectively. The short-term lenvatinib protocol could be superior to the long-term protocol from the point of view of avoiding the risk of tumor vessel starvation while minimizing adverse events. Although the follow-up durations were short, there have been no recurrences so far. Further prospective studies are mandatory in order to show the efficacy of short-term lenvatinib administration prior to cTACE.

## Data Availability

The datasets generated and/or analyzed during the current study are available from the corresponding author on reasonable request.
